# Higher Precision in Pointing Movements of the Preferred vs. Non-Preferred Hand Is Associated with an Earlier Occurrence of Anticipatory Postural Adjustments

**DOI:** 10.3389/fnhum.2016.00365

**Published:** 2016-07-18

**Authors:** Carlo Bruttini, Roberto Esposti, Francesco Bolzoni, Paolo Cavallari

**Affiliations:** Human Motor Control and Posture Laboratory, Human Physiology Section of the DePT, Università degli Studi di MilanoMilan, Italy

**Keywords:** motor control, posture, APAs, handedness, precision, human

## Abstract

It is a common experience to exhibit a greater dexterity when performing a pointing movement with the preferred limb (PREF) vs. the non-preferred (NON-PREF) one. Here we provide evidence that the higher precision in pointing movements of the PREF vs. NON-PREF hand is associated with an earlier occurrence of the anticipatory postural adjustments (APAs). In this aim, we compared the APAs which stabilize the left or the right arm when performing a pen-pointing movement (prime mover flexor carpi radialis (FCR)). Moreover, we analyzed the elbow and wrist kinematics as well as the precision of the pointing movement. The mean kinematics of wrist movement and its latency, with respect to prime mover recruitment, were similar in the two sides, while APAs in triceps brachii (TB), biceps brachii (BB) and anterior deltoid (AD) were more anticipated when movements were performed with the PREF than with the NON-PREF hand (60–70 vs. 20–30 ms). APAs amplitudes were comparable in the muscles of the two sides. Earlier APAs in the preferred limb were associated with a better fixation of the elbow, which showed a lower excursion, and with a less scattered pointing error (PREF: 10.1 ± 0.8 mm; NON-PREF: 16.3 ± 1.7). Present results suggest that, by securing the more proximal joints dynamics, an appropriate timing of the intra-limb APAs is necessary for refining the voluntary movement precision, which is known to be scarce on the NON-PREF side.

## Introduction

Anticipatory postural adjustments (APAs) are commonly defined as unconscious muscular activities aimed to counterbalance the perturbation caused by the primary movement. In this respect, they have been shown to ensure whole-body balance (Massion, 1992; Bouisset and Do, 2008) and to stabilize body segments (Patla et al., 2002). They are also involved in initiating the displacement of the body center of mass when starting gait (Brenière et al., 1987) and forward reaching (Stapley et al., 1998, 1999). The APAs originate from a feed-forward command (Belen’kĭ et al., 1967; Aruin and Latash, 1995), tailored on several kinematical aspects of the primary movement. Within a few trials, the central nervous system (CNS) is able to adapt APAs to changes in the desired movement speed (Shiratori and Aruin, 2007; Esposti et al., 2015), amplitude of motor action (Aruin and Shiratori, 2004) and the expected mass of the moving segment (Friedli et al., 1984; Toussaint et al., 1998). APAs have been first illustrated in movements that involve a relatively large mass, such as a shoulder flexion, that would produce a backward displacement of the center of mass projection on the ground (Bouisset and Zattara, 1987). Such perturbation may lead to the loss of the whole body equilibrium (Hess, 1943; Bouisset and Zattara, 1987); therefore, in order to counteract it, the recruitment of the prime mover muscles is normally preceded by *inter-limb APAs* in lower limbs, hips and trunk, which in turn induce a forward displacement of the center of mass, preventing falling.

More recently, Aoki (1991) reported that a pattern of postural activity in arm muscles also precedes voluntary wrist movements (*intra-limb APAs*), and that this pattern is related to the movement direction in space, just as similar to the inter-limb APAs described above. In this path, Caronni and Cavallari (2009) reported that also when voluntarily flexing a single segment of tiny mass, such as the index-finger, an intra-limb APA chain develops in several upper-limb muscles, to stabilize the *segmental* equilibrium of the whole arm. Similarly to the APAs preceding wrist flexion, the APA pattern associated to a finger flexion changes according to the direction of the focal movement. Indeed, with the prone hand, both wrist and index-finger flexions are preceded by an excitatory burst in triceps brachii (TB), while biceps brachii (BB) and anterior deltoid (AD) show a concomitant inhibition. Instead, with the hand supine, the opposite occurs: BB and AD show excitatory APAs, while TB undergoes a concomitant inhibition. From these last data, an additional role of APAs may be envisaged: besides maintaining the whole-body equilibrium, it seems that APAs may be important in refining voluntary movement precision. In fact, according to the results obtained in a four-joint software mechanical model and confirmed by an electrical stimulation of the median nerve, Caronni and Cavallari (2009) suggested that intra-limb APAs not only guarantee the maintenance of the arm posture, but may be also very important in controlling the trajectory and the final position of the moving segment. However, up to now, no direct evidence of such a conclusion has been reached when dispatching a voluntary motor command.

To shed further light on the relationship between APAs and movement precision, we studied pointing movements performed by flexing the wrist while holding a digitizer pen, so as to ascertain whether the well known imprecision of the non-preferred (NON-PREF) vs. preferred (PREF) arm (Woodworth, 1899) leading to smaller pointing errors with the PREF limb (Roy et al., 1994; Ypsilanti et al., 2009), may be correlated to a worse control of APAs. Considering that APAs are scaled according to the mass of the moving segment and given that the two hands have a similar mass, it would be surprising to observe differently structured APAs accompanying similar movements of the two sides. On the other hand, a different APAs programming between the two sides, e.g., in timing and/or amplitude, would demonstrate that APAs are essential in refining movement precision.

## Materials and Methods

Experiments were carried out in 13 adult healthy volunteers (4 females); mean age 31.7 ± 9.4 years. All subjects were right-handed, as confirmed by their scores on the 10-item version of the Edinburgh Handedness Inventory (Oldfield, 1971). The procedure was conducted in accordance to the Declaration of Helsinki. All subjects provided written informed consent; none of them had any history of orthopedic or neurological diseases. No ethical approval was required because the experimental procedure was non-invasive, did not require any drug administration and was carried out on healthy volunteers.

### Experimental Procedure

Subjects sat on a chair with both arms along the body, elbows flexed at 90°, the wrists prone and in axis with the forearm. The moving wrist was kept unsupported and slightly extended, with the dorsum of the first metacarpo-phalangeal joint in contact with a proximity switch (CJ10–30GK-E2, Pepperl+Fuchs^®^, Mannheim, Germany). Subjects were explicitly asked to keep their back supported, the arm/forearm still and to look at the target during the experiment, to be aware of their movement performance. Subjects were asked to hold a digitizer pen with the most natural pinch grip and to briskly flex their wrist so as to point to the target with the pen tip as quick and precise as possible (pen-pointing). The target consisted in two orthogonal lines drawn on a white paper, taped on the pen tablet (Intuos Pen and Touch small, Wacom^®^, Saitama, Japan; active tablet area 152 × 95 mm; pen weight 12.5 g). The lines were 1 mm thick × 2 cm long, so that the target center was clearly visible. The chair was height adjustable, while the proximity switch and the pen tablet were screwed on articulated arms (Manfrotto 143 MAGIC ARM^®^ + 035 Superclamp Kit, Manfrotto^®^, Cassola, Italy), so as to adapt to the different body dimensions of the subjects. In the initial position, the pen tip was held approximately 6–7 cm above the cross.

Each pen-pointing movement was self-paced and performed after an acoustic signal. The time between the beep and the movement onset varied according to the subject will; this procedure was adopted to exclude any reaction time.

In each experiment, two sessions of 60 pen-pointing movements were performed, one with each hand (PREF vs. NON-PREF); each session was divided into four sequences of 15 movement trials. The 15 trials were accomplished in a temporal window of about 2 min, and then the subject had time to rest (about 3 min) before undergoing a new sequence. Subjects never complained about fatigue.

At the end of each session, the EMGs of the BB, TB and AD muscles of each side were separately evaluated during a maximal press against a fixed support, which changed according to the tested muscle. The upper limb was kept in the same posture adopted during the pen-pointing experiments, so as not to alter the positioning of the recording electrodes with respect to the muscles. Subjects were thus asked to push as hard as possible upon a fixation point and maintain the push for 5 s. When testing the AD, the fixation point was positioned in front of the arm, at the level of the cubital fossa, and subjects had to push forward against it by trying to flex the arm at the shoulder. When testing the TB, the fixation point was positioned under the wrist and subjects had to push downward against it by trying to extend the forearm at the elbow. When testing the BB, the fixation point was positioned over the wrist and subjects had to push upward against it by trying to flex the forearm at the elbow.

### Movement and EMG Recordings

The onset of the wrist flexion was monitored by the proximity switch. Flexion-extension of wrist and elbow joints was recorded by strain-gauge goniometers (mod. SG65 and SG110 respectively, Biometrics Ltd^®^, Newport, UK) taped to the skin over the respective joint. Angular displacements were DC amplified (P122, Grass Technologies^®^, West Warwick, RI, USA), A/D converted at 2 KHz with 12 bit resolution (PCI-6024E, National Instruments^®^, Austin, TX, USA) and stored. Goniometer calibration was undertaken before each experimental session.

Pairs of pre-gelled surface electrodes, 24 mm apart, (H124SG, Kendall ARBO, Tyco Healthcare, Neustadt/Donau, Germany) were used to record the EMG signal from the prime mover flexor carpi radialis (FCR) and from some of the ipsilateral postural muscles: BB, TB and AD. A good selectivity of the EMG recordings was achieved both by a careful positioning of the electrodes over the skin covering the muscle belly and by checking that the activity from the recorded muscle, during its phasic contraction, was not contaminated by signals from other sources. EMG was AC amplified (IP511, Grass Technologies^®^, West Warwick, RI, USA; gain 2–10 k) and band-pass filtered (30–1000 Hz, to minimize both movement artifacts and high frequency noise). Goniometric and EMG signals were A/D converted at 2 kHz with 12-bit resolution (PCI-6024E, National Instruments^®^, Austin, TX, USA), visualized online and stored for further analysis.

The position of the subject was always visually controlled by the experimenter, who also evaluated the amplitude and duration of each pointing movement by looking at the wrist angle trace on the computer screen, so as to remind the subject to speed-up the movement, if necessary.

### Data Analysis

For each tested hand, the 60 EMG traces of the prime mover and those simultaneously recorded from the postural muscles were digitally rectified and integrated (time constant: 25 ms). All the EMG and goniometric traces were then averaged in a fixed temporal window: from −1000 to +300 ms with respect to the onset of the FCR EMG, identified by a software threshold set at +2 SD of the initial excitation level (from 1000 to 500 ms prior to movement onset).

On each experiment, latency and amplitude of the postural activity was measured off-line on the averaged traces, after subtracting from them the initial excitation level. Since the initial EMG level represented the tonic activity required to keep the upper limb in the experimental position, the APAs superimposed on it and should therefore be measured as changes (either positive or negative) in postural muscles activity. Also the peak-to-peak amplitude of wrist and elbow movements were measured off-line on the averaged traces, after subtracting their initial level for illustration purposes. The EMG onset in each postural muscle was identified by a software threshold set at ±2 SD of the initial excitation level, and visually validated. Latency of the APA was referred to the FCR EMG onset, with negative values indicating a time-advance. In order to measure the amplitude of each APA, the rectified EMG was first integrated from the APA onset to the movement onset. The resulting value was then normalized to the corresponding reference value, which was calculated as the mean value of the rectified EMG during the 5 s maximal press multiplied by the APA duration. APA amplitudes (normalized EMGi) were thus expressed in % of the reference value.

The peak-to-peak angular displacement of the elbow joint was measured from the onset of wrist flexion, signaled by the proximity switch, to the moment when flexion started to be braked, i.e., when wrist acceleration zeroed. The time of first contact between the pen tip and the tablet signaled the movement end; the distance between the pen-tip position and the target center at that moment being the pointing error. APA latencies and APA amplitudes (absolute values) were compared by a repeated measures analysis of variance (ANOVA) with factors *muscle* (BB vs. TB vs. AD) and *side* (PREF vs. NON-PREF). All other PREF vs. NON-PREF comparisons were performed by using paired *t*-tests. Statistical significance was set at *p* < 0.05.

## Results

**Figure 1:**
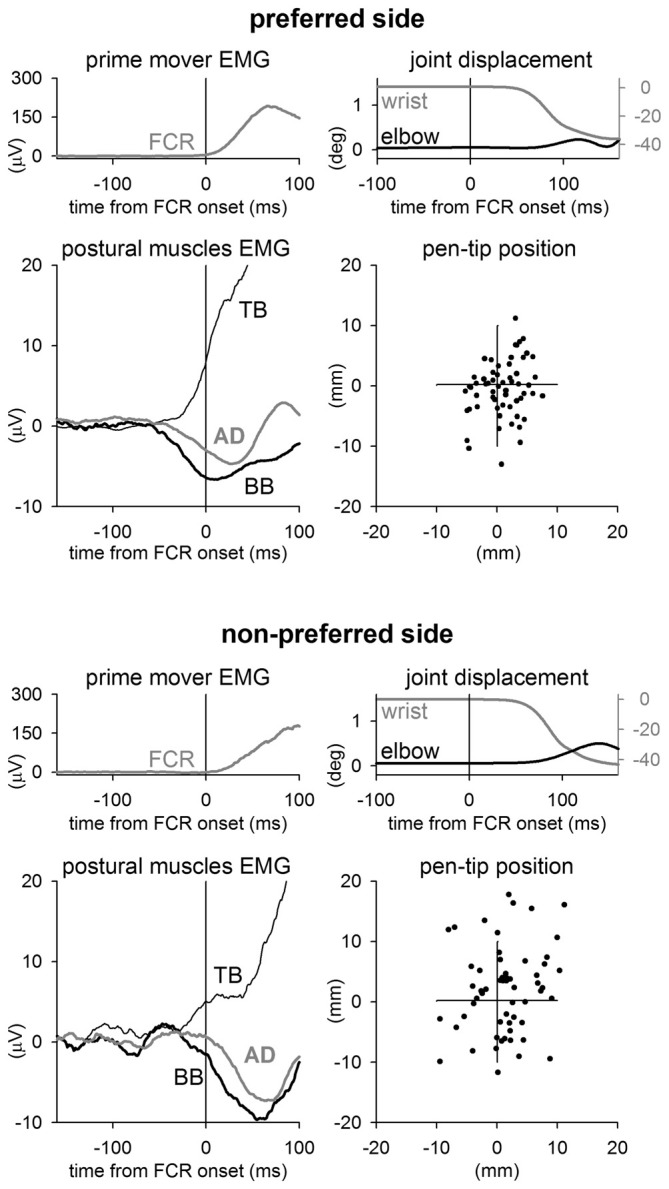
**Sample recordings from a representative subject.** Rectified and integrated average recordings of EMG in Flexor Carpi Radialis (FCR, prime mover), Biceps Brachii (BB), Triceps Brachii (TB), Anterior Deltoid (AD), together with wrist flexion, elbow excursion and ensuing final position of pen-tip. Time 0 = FCR onset. When pointing with the preferred wrist (upper panels), the right elbow equilibrium was preserved thanks to excitatory APAs in TB and inhibitory APAs in BB and AD, which precede the FCR activation by about 65 ms. In the non-preferred side (lower panels) APAs were delayed, indeed they advanced the prime mover onset by just 20–30 ms. This was associated with an increase of the elbow excursion during the wrist movement. The effect that the different APA timing and the associated elbow stabilization had on the pen-tip final position is shown on the right-lowermost panels.

When pointing while holding a pen with the PREF hand the FCR muscle activation was preceded by clear inhibitory postural adjustments in BB and AD muscles, and by an excitatory postural adjustment in TB (Figure 1, representative subject). This APA pattern preceded wrist flexion of about 60 ms. Instead, when the same subject pointed with the NON-PREF hand, APAs showed a similar pattern (excitation in TB and inhibition in BB and AD), but were clearly less anticipated. This change in the APA timing was associated to an increased elbow angular displacement during the movement, with respect to the PREF side. Thus, thanks to a better stabilization of the proximal joint, the representative subject was more precise in PREF than in NON-PREF. Indeed, this is apparent when comparing the final position of the pen-tip in the two sides.

**Figure 2:**
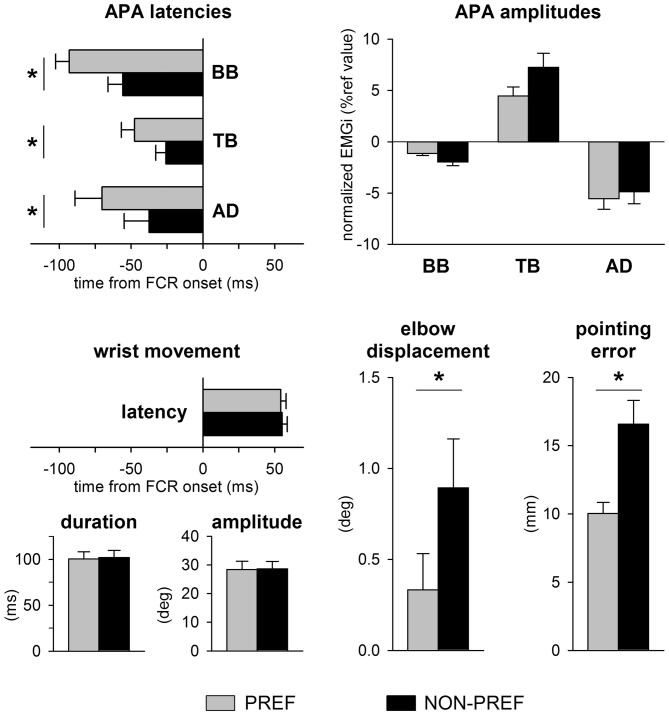
**Data from all subjects.** Top panels show APAs latencies and amplitudes in TB, BB and AD of the preferred (PREF, gray) and of the non-preferred (NON-PREF, black) sides. Bottom panels report the latency, duration and amplitude of the wrist movement, as well as the elbow displacement and the pointing error on each side. Changes in APA latency were associated to a significant increase of the elbow displacement and pointing error. Time 0 = EMG onset in the prime mover Flexor Carpi Radialis (FCR). Mean values ± SEM. *p < 0.05.

Figure 2 illustrates the mean latency and amplitudes of APAs, the average latency, duration and amplitude of the wrist movement, as well as the average elbow displacement and pointing error in the whole sample. Note that the latency, amplitude and duration of wrist movement were at all similar in the PREF vs. NON-PREF hand (latency: *t*_(12)_ = 0.19, *p* = 0.84; duration: *t*_(12)_ = 0.32, *p* = 0.75; amplitude: *t*_(12)_ = 0.17, *p* = 0.86). Despite the invariance of average kinematics of the focal movement, the excitatory APA in TB and inhibitory APAs in BB and AD were delayed of about 20–30 ms in NON-PREF with respect to PREF, the time shift being similar in all muscles. Indeed, two-way ANOVA only found a main effect of *side* (*F*_(1,12)_ = 18.43, *p* = 0.001), with no effect of *muscles* (*F*_(2,24)_ = 2.90, *p* = 0.075), nor *interaction* (*F*_(2,24)_ = 0.71, *p* = 0.50). The absolute values of APA amplitude were different in the three muscles but did not show any significant PREF vs. NON-PREF difference. Indeed, two-way ANOVA only found a main effect of *muscle* (*F*_(2,24)_ = 16.65, *p* < 0.0001; Tukey *post hoc* revealed that APA was lower in BB than in TB and AD, with no difference among the latter two), with no effect of *side* (*F*_(1,12)_ = 1.23, *p* = 0.29) nor *interaction* (*F*_(2,24)_ = 2.05, *p* = 0.15).

When pointing with the NON-PREF hand, the delay of the APA chain was associated to a greater displacement of the elbow joint with respect to the PREF side (*t*_(12)_ = 3.68, *p* = 0.0035) and to a larger and more scattered pointing error (*t*_(12)_ = 5.18, *p* < 0.0002).

## Discussion

This article illustrates an asymmetry in the temporal organization of the intra-limb APAs that stabilize the arm when producing a pen-pointing movement, performed by flexing one or the other wrist. Indeed, when pointing with the NON-PREF side, the APA chain in BB, TB and AD was less anticipated with respect to FCR recruitment. Considering that the mechanical perturbations induced by the focal movements were similar in the two sides, as witnessed by the similarities of wrist kinematics, the change in the APA timing was associated to the less efficient fixation of the elbow joint, which led to the reduced precision of the pointing movement. Therefore, we propose that the increased precision of the PREF hand stems from a more precise tailoring of APAs timing in that side.

### Considerations About APA Amplitude Measurements

Given the difference in APA latency, statistics about APA amplitude should be interpreted carefully. In fact, they are based on data which do not represent comparable portions of the feedforward postural commands in the two sides. The onset of this command clearly produces the APA onset, but its end may occur either before or after the movement onset. As an example, the AD inhibitory APA, illustrated in Figure 1, seems to decay before wrist flexion in the PREF side while it decays after such movement in the NON-PREF side. Therefore, the APA amplitude in the PREF side fully quantifies the expression of the feedforward command, but it represents only a fraction of it in the NON-PREF side. In other words, the feedforward postural command does not necessarily end within the APA but may as well continue during the movement, when the simultaneous postural adjustments (SPAs) occur. In this case, SPAs become expression of a compound feedforward-feedback signal, in which it is impossible to identify the end of the feedforward component.

To overcome this problem, it could have been possible to choose a fixed time-window, ending before the movement onset. However, amplitude measurements would have been again inconclusive. Indeed, if the time window had ended before the APA peak, the measurement would have approximated the slope (rate of change) of the feedforward command, not its real amplitude. If instead the time-window had included the APA peak but not its decay, the measurement would have mixed-up amplitude and slope, in variable proportions.

In conclusion, the absence of statistical differences between the APAs amplitudes in the two sides does not grant that the respective feedforward commands were also comparable. Instead, since the APAs onset is the direct expression of the onset of the feedforward command, the observed change in latency grants for a different the temporal organization of the APAs.

### APAs Accompanying Preferred and Non-Preferred Limb Movements

Literature about *lateralization* of motor control shows a greater skill in the PREF vs. NON-PREF side for arm, wrist and finger movements (Todor et al., 1982). Substantial differences were illustrated in the coordination of muscular recruitment and intersegmental torques in the two sides, which in turn implied a more efficient strategy for the dominant (PREF) arm (Sainburg and Kalakanis, 2000; Sainburg, 2014) as well as a right-hand superiority in task such as throwing (Watson and Kimura, 1989), and a superiority in control of body stabilization during rapid step initiation with the PREF-leg (Yiou and Do, 2010). In particular, Bagesteiro and Sainburg (2002) illustrated that the more efficient strategy for the dominant arm was associated to different EMG profiles and therefore, they suggested that manual asymmetries result from differences between the two sides in controlling the effects of limb dynamics (see also Oliveira and Sanders, 2015).

Little is known instead about APAs and lateralization, since the large majority of studies on APAs have been conducted on voluntary movements performed with the PREF hand/side. Only a few studies have addressed the lateralization question (e.g., Teyssèdre et al., 2000; Mezaour et al., 2009; Yiou and Do, 2010). Teyssèdre et al. (2000), as an example, investigated whether lateral preference for one upper limb could involve side differences in APAs of postural muscles, showing an earlier occurrence of APAs for pointing movements performed with the PREF upper limb, and concluding that lateral preference is associated with postural laterality.

On the other hand, with regard to the central organization of the postural control, it has been recently shown that the left hemisphere in right-handers was more involved in the predictive control of the body and the consequent environmental dynamics. Instead, the right hemisphere of the same subjects was more involved during the deceleration phase of motion, as an impedance control mechanism to terminate the movement (Yadav and Sainburg, 2014). Consequently, it could be argued that the enhanced predictive control of body and environmental dynamics, driven by the left hemisphere, led to the more efficient anticipatory postural strategy, as we observed in the PREF side of our right-handed subjects. Recently, by studying long-latency stretch reflexes as a mechanism that permits the postural control, it has been suggested that handedness affects more the feedforward strategies than those based on sensory feed-back (Walker and Perreault, 2015).

In summary, the *lateralization* of APAs observed in the present study is in accordance with all the above described articles and provides novel evidence in favor of a functional linkage between the timing of intra-limb APAs, the resulting stability of elbow joint and the ensuing precision of pen-pointing movements.

### APAs and Precision

According to shooting coaches and athletes, good postural balance is a vital component of a successful shooting performance. During bipedal standing, top-level rifle shooters stabilized their whole body balance better than naive shooters (Aalto et al., 1990); the capability to reduce the oscillation, especially in the last few seconds before pulling the trigger, expresses the better control of posture in athletes and was associated with a better shooting performance (Era et al., 1996; Mononen et al., 2007). Ypsilanti et al. (2009) illustrated a better stabilization of the center of pressure (CoP) and an improvement in the movement precision for pointing movements performed with the PREF than NON-PREF upper-limb, but did not provide results regarding the APAs chain. More recently, Furuya et al. (2011) demonstrated that professional pianists tended to play using less muscular activity and to take greater advantage of shoulder joint rotation during a keystroke than did novice.

The idea that the precision of a voluntary movement relies on proper APAs was first proposed for what concerns the *inter-limb* APAs. To the best of our knowledge, this idea was forwarded by studies that analyzed the linkage between APAs and movement precision in pointing to targets of different sizes (Nana-Ibrahim et al., 2008; Bertucco and Cesari, 2010). However, these results might be an outcome of the relationship between APAs and intended movement speed (Shiratori and Aruin, 2007; Esposti et al., 2015), since the speed of voluntary movements varies as a function of the target width, according to the Fitts (1954) law. In order to get rid of the possible bias due to changes in movement speed, Caronni et al. (2013) studied inter-limb APAs during an upper limb pointing movement before and after donning prismatic lenses, which are known to shift the binocular eye field and cause the subject to miss the target (Redding et al., 2005). By using this experimental paradigm, these authors showed that, despite similar kinematics of the focal movement, pointing errors occurred when lower limb APAs were out of proportion with respect to the recruitment of prime mover muscles in the shoulder. It has been also recently demonstrated that training induce improvement in the correct tailoring of the APA chain with respect to the prime mover recruitment, both in young adults (Kanekar and Aruin, 2015) and elderly (Kubicki et al., 2012; Aruin et al., 2015). Thus, the increased precision of voluntary movement observed after training (Hamman et al., 1995; Yang et al., 2013) might be partly due to a more appropriate tuning of APAs on the prime mover recruitment. The linkage between APAs and movement precision was also suggested for *intra-limb* APAs by Caronni and Cavallari (2009). Indeed, these authors showed that when simulating an index-finger flexion using a software mechanical model of the arm, a clear disturbance of both focal movement and upper-limb posture was observed, with relevant changes at wrist and elbow level. In the model, the only way to prevent these effects was to block all segments but the finger, preventing the proximal joints from rotating (*fictive* APAs). Since this observation derived from a very simplified system, Caronni and Cavallari (2009) also looked for a more realistic model: a finger tap was thus evoked in a real arm by electrical stimulation of the median nerve. This experiment showed similar recordings to those predicted by the software mechanical model. However, both the software stimulation and the electrically evoked tap did not faithfully represent the physiological pointing movement, since in the two cases no voluntary command was generated. Thus, considering that the experimental paradigm used in the present article involved subjects who voluntarily performed a pointing movement, present results are helpful for completing the framework originally proposed by Caronni and Cavallari (2009).

### Further Considerations on the Motor Program for APAs and Prime Mover Recruitment

Present data should be discussed in the framework of the suggestion of a *shared motor command* for both APAs and prime mover recruitment (Aruin and Latash, 1995; Stapley et al., 1999; Leonard et al., 2011; Caronni et al., 2013). In particular, it was recently demonstrated that the intra-limb APAs stabilizing the arm when producing a brisk index-finger flexion were still present under an ischemic block of the forearm that suppressed the prime mover EMG, the finger movement and the related mechanical perturbation. Indeed, APAs remained tailored to the intended movement, i.e., to the expected perturbation, even after 60 movement trials in which that perturbation did not occur (Bruttini et al., 2014). Furthermore, in the same article, it was illustrated that intra-limb APAs were strongly attenuated when adding a fixation point to the wrist, i.e., closer to the voluntary moving segment (index-finger), a result that agrees with arm-pull experiments in standing subjects (Cordo and Nashner, 1982; Dietz and Colombo, 1996). Altogether, those results support the idea that the recruitment of postural and prime mover muscles should be driven by a *shared motor command*, according to a well-acquired pattern, which drives the muscular chain starting from the fixation point(s) and including the moving segment. Indeed, the shared motor command theory states that the central command to postural muscles and prime movers is “unique”, so that the postural and voluntary components cannot be decoupled. However, this does not imply that a given voluntary command is always coupled to the same postural command. Thus, the observed time difference in postural commands between the PREF and NON-PREF limb can simply be the expression of a *badly* matched timing within the shared motor command (postural vs. focal components) in the NON-PREF side, which in turn may simply result from the reduced motor experience in that side. This latter interpretation seems the one to be PREF, also considering that a similar timing disruption was observed in some pathological studies, in particular for what concerns cerebellar dysfunction. Indeed ataxic patients, who typically display dysmetria (i.e., the inability to precisely reach a given target), showed a temporal disruption of intra-limb APAs both during finger flexions (Bruttini et al., 2015) and in the bimanual unloading task (Diedrichsen et al., 2005). Data from these pathological studies further strengthen the linkage between APA timing and movement precision we observed in the present study. Considering that physical exercise enhances APAs (Kubicki et al., 2012; Aruin et al., 2015; Kanekar and Aruin, 2015) and movement precision (Aalto et al., 1990; Era et al., 1996; Mononen et al., 2007), while a short-term immobilization alters the APAs control (Bolzoni et al., 2012), it is more than probable that training the NON-PREF side would effectively improve APAs timing and movement precision.

Taking into account all these considerations, the classical definition of APA (Massion, 1992) might be extended, for instance, to: motor activities* starting from a fixation point*, aiming to* produce the necessary dynamics* so as to* refine the precision and accuracy of a voluntary movement*, thus implicitly taking into account the perturbation induced by the primary movement.

## Conclusion

Present results showed a lateralization of the intra-limb APAs stabilizing the arm when producing a pen-pointing movement. The APAs delay in movements performed with the NON-PREF hand, in comparison to the recordings of the preferred side, were associated to an impaired stability of the elbow joint, with similar kinematics of the focal movement in the two sides. As a result, the focal movement perturbation caused an increased elbow excursion in the NON-PREF upper-limb, eventually leading to the diminished movement precision on that side. These data strengthen the idea that the APA chain is essential for an appropriate stabilization of the joints involved in the posture-focal chain and therefore, allows refining the precision of the focal movement.

## Author Contributions

All authors contributed in conceptualizing and designing the experiment, acquiring and analyzing the data, interpreting the results and writing the article. All authors approved the final version and agree to be accountable for all aspects of this work.

## Conflict of Interest Statement

The authors declare that the research was conducted in the absence of any commercial or financial relationships that could be construed as a potential conflict of interest. The reviewer CT and handling Editor declared their shared affiliation, and the handling Editor states that the process nevertheless met the standards of a fair and objective review.
